# *Clinical Orthopaedics and Related Research, The Bone & Joint Journal,* the *Journal of Orthopaedic Research,* and *The Journal of Bone and Joint Surgery* Will Not Accept Clinical Research Manuscripts Previously Posted to Preprint Servers*

**DOI:** 10.2106/JBJS.18.01215

**Published:** 2019-01-02

**Authors:** Seth S. Leopold, Fares S. Haddad, Linda J. Sandell, Marc Swiontkowski

Fifty years ago, *The New England Journal of Medicine (NEJM)* clarified a policy about what we now would call redundant or duplicate publication. The Editor-in-Chief of *NEJM* at the time, Franz J. Ingelfinger, made it clear that (apart from a few exceptions) his journal would not publish research that had been submitted to any other journal or published in the media^1^. What has since come to be known at *NEJM* and elsewhere as the “Ingelfinger rule” has endured, largely unchanged^2,3^, to this day.

It also has been the explicit policy of *The Bone & Joint Journal (BJJ), Clinical Orthopaedics and Related Research (CORR), The Journal of Bone and Joint Surgery (JBJS),* and the *Journal of Orthopaedic Research (JOR)* not to accept research papers submitted or published elsewhere either as a whole or in part. We are not alone in this regard. The leading international bodies whose standards of scholarly publishing we seek to adhere to, including the Committee on Publication Ethics (COPE) and the International Committee of Medical Journal Editors (ICMJE), both consider (again, with a few exceptions) prior submission or publication of work sent to a journal for review to be an ethical and practical problem; according to COPE, it is grounds for retraction of a published paper^4^, and the ICMJE lists numerous harms that it can cause, including, but not limited to, “inadvertent double-counting of data or inappropriate weighting of the results of a single study, which distorts the available evidence.”^5^

But science and changes within the world of scholarly publication march on, and perhaps restrictions on prior publication are no longer necessary or even reasonable. Certainly, that is the viewpoint of those who espouse the development of medical preprint servers^6-8^, although we do not agree with them for reasons that we will explain.

For those who are unfamiliar, a preprint server allows authors to make public full-length versions of complete manuscripts that have not yet passed peer review. Preprint servers offer the benefits of durability, speed of posting, and easy access by the public. Other potential advantages include the ability for authors to establish precedent (“we are the first to report…”), to receive feedback on the work from other scientists, and to disseminate results without barriers such as journals’ subscription paywalls or the delays associated with peer review. Advocates of preprint servers feel that they can help mitigate positive-outcome bias and that they increase transparency and data sharing^7,8^, the latter being a requirement of important funding bodies such as the National Institutes of Health^9^ and the Wellcome Trust^10^. Perhaps for these reasons, several major funding bodies have expressed public support for the development of preprint servers^11^.

Preprint servers have been an accepted part of the scholarly publishing landscape in the physical sciences and mathematics for many years, and a preprint server in the biological sciences—bioRχiv (the last four letters being a typical preprint server naming convention; pronounce them “archive”)—has posted year-over-year increases in usage and now publishes clinical research^12,13^, including some on orthopaedic topics. A preprint server specifically for medical research, medRχiv, now is being developed by a partnership consisting of the Yale University Open Data Access (YODA) Project, Cold Spring Harbor Laboratory, and *BMJ*^14^. Others may be on the way^15^. While some well-respected journals, including *JAMA,* are staying on the sidelines or actively discouraging authors from posting to preprint servers^16^, dozens of other publishers and journals (including *The Lancet*^17^ and of course *BMJ*) are on board with the concept of preprint servers.

Despite the high-octane support already behind the unproven concept of medical preprint servers, we believe that there are fundamental differences between the communities served by the existing preprint servers in mathematics, physics, and biology and the patients whose lives may depend on high-quality, peer-reviewed biomedical research. We believe that the benefits proposed by advocates of medical preprint servers can be better achieved in other ways, and that medical preprint servers pose serious health and safety dangers to the patients for whom are supposed to be caring.

We have many concerns about medical preprint servers and their potential to cause far more harm than benefit. Five of the most important are:

1. **Preprint servers may be perceived by some (and used by less scrupulous investigators) as evidence even though the studies have not gone through peer review; the public may not be able to discern an unreviewed preprint from a seminal article in a leading journal.** We are concerned that publishing in a preprint server may be a self-serving move by individuals with secondary-gain incentives and by those whose work is unlikely to withstand serious scrutiny by peer-reviewed journals. These individuals may benefit from the likelihood that most researchers in academic medicine have neither the time nor the inclination to peruse these servers and offer unsolicited comments on what may be junk science. But content on these servers still can be referenced on web sites, cited in peer-reviewed publications, and used to promote treatment approaches to unsuspecting—and sometimes desperate—patients. The weaker the idea, the greater the incentive for the unscrupulous to use preprint servers in medicine or surgery. Although content on many preprint servers is identified as not having been peer reviewed, the watermarks indicating this often are small or obscure.

2. **It seems unlikely that the kind of prepublication dialogue that has taken place in other academic disciplines (such as mathematics and physics) will take place in medicine or surgery because the incentives are very different.** Even high-quality journals in medicine and surgery struggle to attain and retain the best peer reviewers, especially in some of the smaller specialties where the reviewer pools are finite; in fact, whole industries have sprung up in an effort to address this problem^18^. This problem exists despite journals’ abilities to offer incentives: subscriptions, recognition by name, continuing medical education credit, and an “item” on one’s curriculum vitae that may help gain academic promotion and connections within the specialty. It is hard to imagine any serious dialogue on medical preprint servers since there is no incentive for people to spend their time offering unsolicited critiques of the material posted there. Data support this contention; even on an established biomedical preprint server, only 10% of papers received comments posted to the site^13^; although more recent unpublished data about that same server suggest that the percentage has increased to about 25%^19^, that still leaves the large majority of papers circulating with claims entirely unvetted by anyone apart from the authors themselves, and with no editorial oversight. Also, many of the preprints that have received some comments are in fact still largely unvetted; there is a world of difference between a few comments posted on a preprint and the kind of thorough peer review that journals like *BJJ, CORR, JOR,* and *JBJS* provide.

3. **Preprint servers may lead to 2 competing, and perhaps even conflicting, versions of the “same” content being available online at the same time, which can cause (at least) confusion and (at most) grave harm**. A typical article undergoing revision in our journals receives dozens of reviewers’ comments that call for modifications or clarifications, and 80 to 100 editors’ queries in the margins of the edited manuscript that require substantive changes. However, someone can search and reference some early version of the “same” work on a preprint server, meaning that, despite all that effort, patients can be harmed by the continued circulation of errors of fact, problematic claims, and unsubstantiated recommendations that were later removed from the “definitive” (that is, peer-reviewed and journal-published) version.

4. **For the vast majority of medical (and especially surgical) diagnoses, a few months of review of a study’s findings do not make a difference; the pace of discovery and dissemination generally is adequate.** For the rare situations where an article reports a time-sensitive discovery (such as a product failure or an unexpected problem with a drug or treatment), all reputable journals provide a fast-track option; certainly, the 4 journals represented here do. The far larger risk is that ideas with insufficient support come out too quickly and gain a hold among practitioners or patients. This is not theoretical: our specialty has seen a surfeit of well-intentioned ideas that have harmed patients, even after being vetted through peer-review and governmental approval processes. It would be far worse if we lowered the bar and presented concepts based on untested data to a wider audience that is unprepared to assess them critically. Preprint servers in medicine may attract the kinds of investigators who wish to get such ideas out, will likely not attract the kind of corrective dialogue needed to fix (or discredit) those ideas, and therefore run a very real risk of harming patients.

5. **There are better ways to mitigate positive-outcome bias and promote transparency, which are two main purported benefits of preprint servers.** These include prospective registration of randomized trials—which all of our journals now require^20^—and good peer review with open-data approaches where needed, which all of our journals currently provide. In contrast, the risks of preprint servers seem to be at odds with some of the principles that they seek to promote. They prioritize pace over careful consideration. They offer no incentives for their already busy readers to make comments, and no incentive for those who are disinclined to do so to correct their posts on those servers. Once ideas have been disseminated on preprint servers, they become immediately available to millions of readers, and the lay public has little ability to discern a manuscript draft on a preprint server from a properly reviewed source. As an important aside, there is nothing particularly “transparent” about a clinician-scientist writing whatever (s)he wishes to, without having to respond to a review process that might modify overstated elements of the message, insist on caveats, identify shortcomings, correct errors, and require full disclosure of any industry involvement in the research. Our reviewers and editors spend considerable time and effort identifying shortcomings in articles that authors did not disclose because they did not support the story that the authors wished to tell. The truth is, first-draft scientific manuscripts often fall quite short in the transparency department, and first drafts are all we are likely to see on medical preprint servers. As far as we know, there is no robust evidence to suggest that preprint servers mitigate positive-outcome bias, as their advocates hope will be the case.

One of us (S.S.L.) visited at length with one of the developers of medRχiv^19^. Another of us (M.S.) was on the board of directors of one of the three organizations that are sponsoring a medical preprint server^21^. We have tried diligently to see their point of view, but after extensive deliberation with our respective boards, we have been unable to convince ourselves that the benefits of preprint servers in clinical orthopaedic research outweigh the potential harm to patients and scientific integrity.

For those reasons and others, *Clinical Orthopaedics and Related Research, The Bone & Joint Journal,* the *Journal of Orthopaedic Research,* and *The Journal of Bone and Joint Surgery* will not accept clinical research manuscript submissions—which we define as research involving human subjects or their medical records—that have been posted to preprint servers prior to submission, and we will withdraw from consideration any papers posted to those servers prior to publication. We exempt from this policy all laboratory research that does not involve human subjects, and we recommit ourselves to offering fast-track publication to those papers that share time-sensitive messages pertaining to patient health or safety.
